# Post-operative Sore Throat: Comparing the Monitored Endotracheal Tube Cuff Pressure and Pilot Balloon Palpation Methods

**DOI:** 10.21315/mjms2019.26.5.12

**Published:** 2019-11-04

**Authors:** Nagappan Ganason, Vanitha Sivanaser, Chian Yong Liu, Muhammad Maaya, Joanna Su Min Ooi

**Affiliations:** 1 Department of Anaesthesiology and Intensive Care, Universiti Kebangsaan Malaysia Medical Centre, Kuala Lumpur, Malaysia; 2 Department of Anaesthesia and Intensive Care, Hospital Kuala Lumpur, Kuala Lumpur, Malaysia

**Keywords:** post-operative sore throat, endotracheal cuff pressure, cuff pressure monitor, pilot balloon, palpation method

## Abstract

**Background:**

Endotracheal tube cuff (ETTc) inflation pressure is usually not regarded as an important aspect during intubation. In this study, we compared measuring ETTc pressure and pilot balloon palpation method in causing post-operative airway complications.

**Methods:**

Two hundred and ninety-two surgical patients requiring intubation were recruited into this prospective, double-blind, randomised controlled study. Group A patients had their ETTc initially inflated, checked by a cuff pressure gauge, recorded and then set to 25 cmH_2_O. Group B patients had their ETTc inflated using the pilot balloon palpation method. Patients were then followed up for post-operative sore throat, hoarseness and cough.

**Results:**

The overall incidence of post-operative sore throat was 39.0% versus 75.3% (*P* < 0.001), hoarseness 6.2% versus 15.1% (*P* < 0.05) and cough 7.5% versus 21.9% (*P* < 0.05) in Group A and B, respectively. Group A patients experienced a significant reduction in the incidence and severity of sore throat up to 24 h post-operatively (*P* < 0.001), hoarseness at the first hour (*P* = 0.004) and cough at first and 12 h post-operatively (*P* = 0.002).

**Conclusion:**

Adjusting the ETTc pressure to 25 cmH_2_O reduces post-operative sore throat, hoarseness and cough compared to pilot balloon palpation method.

## Introduction

Post-operative sore throat (POST) and hoarseness of voice are complications of general anaesthesia requiring tracheal intubation, with reported incidences of sore throat varying from 11% to 48% and hoarseness from 18% to 53% (1). POST may be considered a minor problem that resolves spontaneously after a few days, but it can cause distress and decrease the quality of recovery and patient satisfaction (2). The mechanism is believed to involve erosion of the tracheal mucosa caused by the cuff of a tracheal tube, trauma from tracheal intubation and mucosal dehydration (3).

Cuff inflation after endotracheal intubation is designed to prevent air leakage, ensuring effective ventilation, reducing leakage of inhalational anaesthetic agents and preventing pharyngeal content aspiration. Over-inflation of the endotracheal tube cuff (ETTc) affects blood supply to the tracheal mucosa, which may result in tracheal mucosal ischemia, ulceration, necrosis or tracheoesophageal fistula (4). An endoscopic study on mucosal blood flow by Seegobin and van Hasselt (5) found that when the ETTc pressure exceeded 30 cmH_2_O, the blood flow to the tracheal mucosa began to decrease, and when the pressure approached 50 cmH_2_O, ischaemic injury to the tracheal mucosa occurred. Therefore, many studies on post-intubation airway complications have recommended that ETTc pressure be kept between 20 cmH_2_O and 30 cmH_2_O (6–9).

For brief procedures lasting only a few hours, most clinicians give little attention to the inflation pressure of the ETTc and simply determine the pressure by pilot balloon palpation according to their experience (7, 9). Studies by faculty emergency physicians, anaesthesiologists, anaesthesia residents and critical care unit staff have demonstrated a prevalent inability of these clinicians to accurately determine ETTc pressure using pilot balloon palpation (10). Despite many studies validating the importance of monitoring the ETTc pressure, many experienced anaesthesiology personnel continue to use the pilot balloon palpation method to estimate the pressure, and in many centres, inflation of the ETTc is left to the anaesthetic nurse or technician.

The aim of this study was to compare the incidence and severity of post-operative sore throat in patients when endotracheal cuff pressure was measured and adjusted versus when the conventional pilot balloon palpation technique was used.

## Materials and Methods

Two hundred and ninety-two patients aged 18 years and above with American Society of Anesthesiologists (ASA) physical status I, II or III who were scheduled for elective surgery in the supine position under general anaesthesia requiring oral endotracheal intubation were included in the study. Patients with anticipated difficult intubation, high risk for aspiration, and those undergoing oral and laryngopharyngeal surgery or requiring nasogastric tube or throat pack insertion were excluded.

The patients were randomly assigned into two groups [Group A (measured ETTc pressure) and Group B (ETTc palpation technique)] by the study coordinator using a computer-generated randomised sequence at the operating theatre on the morning of the surgery. The coordinator and the attending anaesthesiologists were not blinded to the group allocation, whereas the patients and the outcome assessors were blinded.

The patients were induced with IV fentanyl 1 mcg/kg–2 mcg/kg, IV propofol 2 mg/kg–2.5 mg/kg and IV rocuronium 0.9 mg/kg and were intubated. The male patients were intubated with an endotracheal tube (ETT) (Idealcare® ETT-cuffed with a high-volume, low-pressure cuff, Malaysia) of 7.5 mm or 8.0 mm in internal diameter, whereas the female patients received a 7.0 mm or 7.5 mm internal diameter ETT. Prior to intubation, all ETTs and stylets were lubricated with lidocaine hydrochloride jelly USP, 2% and all the ETTcs were connected to a three-way stopper to prevent air leakage during measurement. All intubations were performed by an anaesthesiologist or a senior registrar using a C-MAC^®^ Video Laryngoscope (Karl Storz, Germany) with an appropriately sized Macintosh blade.

In Group A, the ETTc was initially inflated by the attending anaesthesiologist using a syringe, and this was followed by a check of the cuff pressure using a cuff pressure gauge (VBM Cuff Pressure Gauge Universal, ref 54-07-000) after securing the endotracheal tube. The initial cuff pressure measured was noted and concealed before setting it to the desired pressure of 25 cmH_2_O. In Group B, the ETTc was inflated by the attending anaesthesiologist with a syringe according to his or her own personal experience using the pilot balloon palpation method. Cuff pressure was not measured in this group. Any patients requiring more than a single attempt for intubation were removed from the study.

Anaesthesia was maintained with sevoflurane or desflurane (MAC 0.9–1.0) using only oxygen/air (fractional inspired oxygen 0.5) with a flow rate of 2 L/min. Standard monitoring procedures were followed, with a standard three-lead ECG, non-invasive arterial blood pressure, pulse oximetry and capnography. Intraoperative analgesia and post-operative nausea or vomiting prophylaxis were given at the discretion of the attending anaesthesiologist.

At the end of the surgery, the posterior pharyngeal wall was gently suctioned. Reversal of the neuromuscular blockade was guided by a nerve stimulator using a train-of-four count. The head end of the operating table was raised to 45° and the ETTc was deflated. The volatile agent was switched off and 100% oxygen was administered. No further stimulation was allowed in order to achieve smooth extubation. The ETT was removed when the patient was fully awake and displaying adequate recovery of neuromuscular function and spontaneous respiration. The times of tracheal intubation and extubation were recorded in both groups, and the range between them was used to define the duration of anaesthesia in the patients.

A medical officer (who was blinded to the study) was assigned to follow up the patients on endotracheal intubation related complications (i.e., sore throat, hoarseness and cough) at 1 h, 12 h, 24 h and 48 h post-extubation, and the assessment was carried out by interviewing the patients. Sore throat was described as present if patients described pain, scratchiness or irritation of the throat, which was further graded using the visual analogue scale (VAS) from 0–10. The severity of the sore throat was regarded as mild (VAS score 1–3), moderate (VAS score 4–7) or severe (VAS 8–10). Hoarseness was described as an abnormal change in voice, such as the voice being breathy, raspy, strained or altered in volume or pitch. Coughs were described as either dry or productive.

The sample size was calculated using the Power and Sample size calculation software version 3.1.2 and was estimated based on information gathered from Jain and Tripathi (8). The power of this study was set at 80%, with an α-value of 0.05; the prevalence of sore throat was 20% in the previous study, and the estimated prevalence was 8% in the experimental subjects (8). Therefore, it was estimated that 130 patients would be required for each group. Anticipating a 10% drop-out rate, 146 patients were eventually recruited into each group.

The data was analysed using the SPSS (Statistical Package for the Social Sciences) software version 23.0. The Chi-square test was used when appropriate to calculate any significant differences across categorical variables (gender, race, ASA physical status and severity of sore throat), and an independent t-test was used to determine any significant differences across continuous variables [age, weight, height, body mass index (BMI) and duration of surgery]. Data with a non-normal distribution were presented as medians. A *P*-value of less than 0.05 was considered statistically significant.

## Results

A total of 292 patients were recruited into this study, with 146 patients in each group. The demographic and intra-operative data of the patients were comparable between the two groups (Table 1).

This study showed that the overall incidence of POST was 39.0% versus 75.3% (*P* = 0.000), hoarseness 6.2% versus 15.1% (*P* = 0.014) and cough 7.5% versus 21.9% (*P* = 0.001) in Groups A and B, respectively. The incidence of sore throat with pain score, hoarseness and cough at the time intervals of 1 h, 12 h, 24 h and 48 h are shown in Table 2. Patients in Group A had a significant reduction in the incidence and severity of sore throat up to 24 h post-operatively (*P* < 0.001). The incidence of hoarseness was significantly different between the two groups only at the first hour (*P* = 0.004) with a marked reduction in Group A but it was not statistically significant beyond the first hour. On the other hand, the incidence of cough in Group A was significantly less up to the first 12 h postoperatively (*P* = 0.002 in both time intervals), but there were no differences beyond that. All of the patients in both groups experienced only mild to moderate post-operative sore throat, which subsided after 24 h, as shown in Table 3. Among the patients in Group A, 35.6% experienced mild pain, compared to 57.5% in Group B, with *P* < 0.001.

In the group in which ETTc pressure was checked and measured after the initial inflation (Group A), it was found that only 82 (56.3%) patients had a cuff pressure within the recommended range of between 20 cmH_2_O and 30 cmH_2_O after the initial inflation. Sixty-three patients (43.2%) had a pressure reading of above 30 cmH_2_O before adjustments were made to 25 cmH_2_O. The highest recorded pressure was 60 cmH_2_O and there was only one instance in which the ETT cuff was underinflated (to 18 cmH_2_O).

## Discussion

In general, POST, cough and hoarseness are common airway complications after intubation. This may be attributed to airway management, female patients, younger patients, surgery for gynaecological procedures and intra-operative use of succinylcholine (2, 6). Higgins et al. (2) found that intra-operative airway management had the greatest influence on the incidence of post-operative sore throat. Out of a total of 5,264 patients who underwent ambulatory surgery under general anaesthesia, they found that patients who were intubated had the greatest incidence (45.5%), followed by patients who had a laryngeal mask airway (17.5%); only 3.3% of patients using the face mask developed postoperative sore throat (2).

Seegobin and van Hasselt (5) measured tracheal mucosal blood flow in adult patients who underwent surgery requiring tracheal intubation using an endoscopic photographic technique and varying the cuff inflation pressures over time. They found that when the ETTc exceeded 30 cmH_2_O, the blood flow in the tracheal mucosa began to reduce, and when the pressure in the cuff approached 50 cmH_2_O for 15 min, ischaemic injury to the tracheal mucosa occurred. They concluded that impaired tracheal mucosal blood flow was an important factor in the tracheal morbidity associated with tracheal intubation and recommended that a cuff inflation pressure of 30 cmH_2_O should not be exceeded. Similarly, in an animal study, Nseir et al. reported that excessive inflation of the ETTc to 50 cmH_2_O for 30 min every 3 h over 48 h in 12 piglets produced high pressure on the tracheal wall, thus affecting the blood perfusion of the tracheal mucosa and resulting in deep mucous ulceration, squamous metaplasia and intense mucosal inflammation (11).

Liu et al. (7) conducted a similar study on 509 patients from four tertiary centres following a similar protocol and found a significant reduction in the incidence of POST, in which 34% of patients who had their EETc pressure measured remained between 15 mmHg and 25 mmHg (20 cmH_2_O–34 cmH_2_O), compared to 44% in the control group, in which the palpation method was used. Their findings were consistent with the findings of this study, in which there was a significant reduction in POST in the group for which the ETTc pressure was adjusted to 25 cmH_2_O. Kaki and Almarakbi found that ETTc inflation guided by pressure volume loop closure resulted in lower ETTc pressure, requiring only 18 cmH_2_O–19 cmH_2_O, in contrast with the ‘just to seal’ technique, requiring 32 cmH_2_O–35 cmH_2_O, hence a lower incidence of POST with the use of volume loop closure (24% versus 38%). This demonstrates that having an objective assessment of the ETTc pressure can significantly reduce the incidence of airway complications (12).

Hoffman et al. (10) conducted a study amongst the licenced faculty of emergency medicine physicians using a tracheal simulation model and revealed that 90% of the physicians over-inflated the ETTc pressure above 120 cmH_2_O and only 22% of these physicians were able to detect over-inflation of ETTc pressure using the pilot balloon palpation method. They concluded that physicians were unable to inflate ETTc to safe pressures or estimate the pressure of ETTc by palpation, and physicians should consider using a device to facilitate safe inflation and accurate measurement of ETTc pressure. Other than the experience of the personnel, the syringe size used to inflate the ETTc also plays a role in over-inflation. In a study by Khan et al. (13), a 10 mL or 20 mL syringe was used to inflate the ETTc, followed by assessment using the palpation method and then further measurement using an aneroid manometer. They found that when using a 20 mL syringe, the anaesthesiologists tended to over-inflate the cuff (86%) more often than when they used a 10 mL syringe (52%).

In this study, 43.2% of the patients in Group A had their ETTc initially inflated to a pressure exceeding 30 cmH_2_O before it was measured and set to 25 cmH_2_O. Therefore, the palpation method was unreliable even in the hands of experienced anaesthesiologists. Hence, ETTc pressure should be measured not only in the operation theatre but in all other settings, including emergency rooms, intensive care wards and remote areas that may require endotracheal intubation to reduce airway complications. We suggest including ETTc pressure measurement as part of the standard monitoring in our local guidelines.

One of the limitations of this study was that the intubation was conducted by many operators. Hence, the force applied on the airway and the technique of intubation may have varied slightly, even though a C-MAC® Video Laryngoscope was used in all patients. In this study, the ETTc pressure in the study group (Group A) was only checked, adjusted and set at 25 cmH_2_O at the beginning of surgery. Even though nitrous oxide was not used, the ETTc pressure should have been monitored throughout the surgery and adjusted accordingly for a better outcome. The other limitation to this study was that the assessment of sore throat was subjective and evaluation of the tracheal mucosa using a fibreoptic bronchoscope was not performed.

## Conclusion

Measuring the ETT cuff pressure and adjusting it to 25 cmH_2_O reduces POST, hoarseness and cough compared to the conventional pilot balloon palpation method.

## Figures and Tables

**Table 1:** Demographic and intra-operative data

	Group A (*n* = 146)	Group B (*n* = 146)	*P*-value
**Age (years)**^a^	44.5 (32.0–56.0)	45.0 (31.0–58.3)	0.642
**Gender**^b^			
Male	65 (44.5)	49 (33.6)	0.072
Female	87 (55.5)	97 (66.4)	
**Race**^b^			
Malay	96 (65.8)	102 (69.9)	0.896
Chinese	22 (15.1)	20 (13.7)	
Indian	24 (16.4)	21 (14.4)	
Others	4 (2.7)	3 (2.1)	
**Weight (kg)**^c^	66.0 (12.5)	64.4 (13.3)	0.275
**Height (cm)**^c^	161.2 (7.8)	160.0 (8.1)	0.219
**BMI (kg/m^2^)**^c^	25.4 (5.1)	24.9 (4.4)	0.397
**ASA**^b^			
1	78 (53.4)	67 (45.9)	0.195
2	68 (46.6)	77 (52.7)	
3	0 (0.0)	2 (1.4)	
**Duration of anaesthesia (min)**^a^	150.0 (114.7–195.0)	150.0 (105.0–195.0)	0.761

a median (IQR),

b number (%),

c mean (SD)

**Table 2:** Incidence of POST, hoarseness and cough

		Group A (*n* = 146)	Group B (*n* = 146)	*P*-value
**Sore throat** a	1 h	57 (39.0)	107 (73.3)	< 0.001
	12 h	16 (11.0)	74 (50.7)	< 0.001
	24 h	5 (3.4)	23 (15.8)	0.001
	48 h	0 (0.0)	1 (0.7)	1.000
	Overall	57 (39.0)	110 (75.3)	< 0.001
**Hoarseness** a	1 h	6 (4.1)	21 (14.4)	0.004
	12 h	6 (4.1)	13 (8.9)	0.153
	24 h	2 (1.4)	1 (0.7)	1.000
	Overall	9 (6.2)	22 (15.1)	0.014
**Cough** a	1 h	10 (6.8)	29 (19.9)	0.002
	12 h	2 (1.4)	15 (10.3)	0.002
	24 h	0 (0.0)	3 (2.1)	0.247
	Overall	11 (7.5)	32 (21.9)	0.001

a Number (%) analysed using Pearson Chi-square test

**Table 3:** Severity of POST

		Group A (*n* = 146)	Group B (*n* = 146)	*P*-value
**1 h**^a^	Mild	52 (35.6)	84 (57.5)	< 0.001
	Moderate	5 (3.4)	23 (15.8)	
**12 h**^a^	Mild	15 (10.3)	70 (47.0)	< 0.001
	Moderate	1 (0.7)	4 (2.7)	
**24 h**^a^	Mild	5 (3.4)	22 (15.1)	< 0.001
	Moderate	0 (0.0)	1 (0.7)	
**48 h**^a^	Mild	0 (0.0)	1 (0.7)	1.000

a Number (%) analysed using Pearson Chi-square test
